# The Tooth Brush and Methods of Cleaning the Teeth*Extract of the report to the American Academy of Periodontology by its committee. Read before the Academy of Periodontology at New Orleans, Oct.. 17, 1919, and reprinted from the "Dental Items of Interest," March 1920

**Published:** 1920-08

**Authors:** Gillette Hayden, Austin F. James, Andrew J. McDonagh

**Affiliations:** Chairman; Special Committee


### THE TOOTH BRUSH AND MEHODS OF CLEANING THE TEETH*

The committee has undertaken the preparation of this report in response to the academy's request that it present a description of a tooth brush of approved design and a method of brushing the teeth which will prove of service to the general practitioner of dentistry in instructing patients in the personal care of the teeth.

The committee has secured from the members of the council opinions resulting from their clinical experiences, and has based its report upon the collective views of these members of the academy.

The effort has been to attempt the standardization of the personal care of the mouth by recommending a brush of an accepted design which may be generally adopted and an efficient method of brushing the gums and teeth which may be universally applied. Both brush and method have been demonstrated by members of the academy to be efficacious and non-injurious.

---

Extract of the report to the American Academy of Periodontology by its committee. Read before the Academy of Periodontology at New Orleans, Oct.. 17, 1919, and reprinted from the "Dental Items of Interest," March, 1920.

In recommending one form of tooth brush and one method of brushing the teeth, the committee does not wish it understood that it discredits *all* brushes of dissimilar design, or *all* methods of brushing the teeth which do not conform to the method outlined, but that it indorses a brush and a method offering the greatest advantages.

The opinion has been unanimously expressed that a brush larger than one and one-half inches in length in the brush head and what is known as the crosswise or mesio-distal stroke method, are both injurious and inefficient.

With this statement of its objects the report is presented to the academy with the announcement that it has been approved by the council, and the request is made that after any necessary modifications have been made, it be accepted and then presented to the National Dental Association with a request for its adoption by that association.

THE TOOTH BRUSH

*For Adults—Brush Head:*

Total length of brush head, $1\frac{1}{4}''$.

Number of bristle tufts in row at neck, at most, 3.

Number of bristle tufts in row at extremity, 2.

Space between rows, $\frac{3}{32}''$ to $\frac{1}{8}''$.

Length of bristle tufts at end, $\frac{1}{4}''$ to $\frac{3}{8}''$.

Length of bristle tufts at neck, $\frac{3}{8}''$ to $\frac{1}{2}''$.

Bristles in tufts should be rounded.

The brushing surface should present a slanting straight line from the neck to the end of the brush head, and it should be convex from side to side.

Bristles should be stiff.

*Handle:*

Should be fairly rigid.

Should be six inches (entire length of handle and brush head).

The neck should be long and slender.

*For Children Over Three Years of Age—Brush Head:*

Total length, 1″.

Number of bristle tufts at neck, 2.

Number of bristle tufts at extremity, 1.

Otherwise, with the exception of a general decrease in size, the child's brush should resemble the adult brush.

*For Children Under Three Years of Age—Brush Head:*

Total length, $\frac{7}{8}$″.

Single bristle tufts set lengthwise of brush head, 5.

Length of bristle tufts at neck, $\frac{5}{16}$″.

Length of bristle tufts at extremity, $\frac{3}{16}$″.

Otherwise, with the exception of a general decrease in size, it should resemble the first child's brush described.

*Methods of Brushing the Teeth*—The American Academy of Periodontology recognizes four effective methods of brushing the teeth, known as:

1. The rolling stroke method.
2. The up and down stroke method.
3. The circular stroke method.
4. The vibratory method.

1. The *rolling stroke method,* which takes precedence over other methods, consists of placing the brush in the mouth bristle ends *up* for the upper teeth, and *down* for the lower teeth, rolling the brush toward the gums and sweeping the bristles over the gums and teeth to their occlusal surfaces.

This motion brushes the buccal and labial surfaces of all the teeth above and below, and the lingual surfaces of the molars and bicuspids in both arches.

The lingual surfaces of the incisors and cuspids are brushed by placing the brush parallel to the tongue, with the bristle ends on the roof of the mouth for the upper teeth and down on the floor of the mouth for the lower teeth, and drawing the brush forward over the gums and teeth to their incisal edges.

The upper and lower jaws are brushed separately.

The occlusal surfaces are brushed with a forward and back and side to side stroke of the brush.

2. The *up and down stroke method* is presented as a practicable compromise between the rolling and the circular stroke methods, and as one whose technique can be readily mastered by many whose personal mouth care might otherwise be less efficient. It consists of placing the brush in the mouth with the bristle ends toward the gums, and while in that position of carrying the brush down over the gums and teeth to their occlusal surfaces and up again for the upper teeth, and the reverse for the lower teeth.

The buccal and labial surfaces of the upper and lower jaws may be brushed separately, or the teeth may be placed edge to edge and both upper and lower jaws brushed at the same time.

The lingual surfaces are brushed in the same way.

The occlusal surfaces are brushed with an in and out and side to side stroke of the brush.

3. The *circular stroke method* consists of placing the brush in the mouth with the bristle ends toward the gums and with a circular motion brushing the gums and teeth. The buccal and labial surfaces of each jaw may be brushed separately or the teeth may be placed edge to edge and both upper and lower jaws brushed at the same time.

The lingual and occlusal surfaces are brushed with the same circular stroke.

4. The *vibratory method* of brushing the teeth consists of placing the brush horizontal to the long axis of the teeth with the bristle ends resting on the tooth surfaces. When pressure is applied to the brush and a very short mesio-distal stroke executed, the bristle ends enter the embrasures between the teeth covered by the brush. The stroke, if it may be called a stroke, is so short that it does not carry the bristles beyond the teeth upon which they rested before the stroke was begun. In this manner all surfaces of the teeth are brushed.

*General Rules for Brushing the Teeth*—Method 1 is a simple and effective method favored by the majority of the members of the academy.

The gums and teeth should be brushed before breakfast and after meals. The most important time to brush is at night.

Those portions of the individual mouth which demand the most care should be brushed first. In general the following rotation may be followed:

Lower jaw—Lingual surfaces of the gums and teeth; buccal surfaces of the gums and teeth; labial surfaces of the gums and teeth.

Upper jaw—Buccal surfaces of the gums and teeth; labial surfaces of the gums and teeth; lingual surfaces of the gums and teeth; occlusal surfaces of both the upper and lower teeth.

The brush should be rinsed frequently in clear water during the brushing.

The tongue should be brushed in the morning.

The brush should be thoroughly rinsed after its use and be placed where it will dry.

*Use of Floss Silk*—Waxed ribbon floss should be used after brushing the teeth and gums. The majority of the members of the academy recommend its use at night. It should be carefully rubbed over the approximal surfaces of the teeth and should not be allowed to injure the gums. The distance between the finger ends when the floss is used should be at all times reduced to the minimum, approximately one-half to three-quarters of an inch. The floss should be stretched taut over the ends of the fingers, or finger and thumb, not around them.

Special care should be given to the approximal surfaces of teeth bounding spaces where one or more teeth are missing. These surfaces may be brushed with a single row bristle brush where the space permits, or they may be cleansed with ribbon floss.

Ribbon floss threaded on a bodkin and passed between the dummy teeth and the soft tissues should be used to cleanse the under surfaces or fixed bridges, and the abutment teeth.

INSTRUCTIONS INTENDED FOR THE LAYMAN

*By the Clock*—Brush your teeth for five minutes, three or four times each day.

1. Before breakfast.
2. After breakfast, or
3. After luncheon.
4. Before going to bed.

Use a *small* brush.

Brush the *gums* and teeth.

Be sure to reach both sides of the rearmost molars.

*To Brush the Teeth*—Place the tooth brush, ends of the bristle *up*, for the upper teeth, between the cheek and the gums; turn the brush toward the gums, sweeping the bristles *down* over the gums and teeth to the cutting edges of the teeth. Reverse for the lower teeth and brush *up*.

This motion brushes the cheek side of all teeth and the tongue side of the back teeth.

Brush the inside of the front teeth by putting the brush, bristles up, on the roof of the mouth for the upper teeth and on the floor of the mouth for the lower teeth and pulling the brush outward over the gums and teeth.

Brush the grinding surfaces of the teeth with an in and out and side to side stroke of the brush.

Rinse the brush in clear water frequently while brushing the teeth, and hang it up to dry when not in use.

A tooth powder paste, or a solution of common table salt may be used as a dentifrice once each day. Use clear water the other times.

DISCUSSION OF THE REPORT

*Dr. Arthur H. Merritt, New York City*—I realize that it is impossible to devise any method of tooth brushing that will meet with the approval of everybody; but there ought to be unanimity of opinion regarding certain fundamental principles. Any method which is at once simple, effective, stimulating and free from injurious influence upon the teeth, should have the approval of this organization and of the dental profession as a whole. It is my opinion that the academy should limit itself to outlining such principles, rather than to go on record as indorsing any one of the several methods which it refers to in its report; to do otherwise is a mistake. I also believe it is a mistake to go on record as recommending the use of waxed silk "if indicated." Is it not always indicated? if so, why not say so? If there is any surface of a tooth which needs care in the prevention of dental caries, it is the approximal surface. Why do these surfaces decay more often than the labial or buccal surface? Simply because they are not kept as clean. They can not be kept clean by any method of tooth brushing that can be invented. At present I know of no way in which they can be reached by the patient except by the use of waxed silk tape. I sincerely hope that this part of the report will be amended before it is approved by the society.

*Dr. Austin F. James, Chicago*—When we attempt the treatment of cavities and proximal surfaces, we find it is almost impossible to do it with floss silk. There is not often a mouth so clean from tapping and methods of that nature that will not have more or less decay if there are bad contact points and spaces into which food can crowd. These should be corrected mechanically.

It is the duty of the dentist to put every tooth surface in a condition so that it can be kept cleaner than the average patient is able to brush his teeth with a brush. It is a definite technique which we should master. It is a part of the work that the dentist should undertake for the patient.

*Dr. H. L. Madison, Burlington, Iowa*—I would like to ask the members whether they instruct their patients to begin brushing the teeth with a dry brush or moisten the brush before they use it. I think that I am getting better results by using a dry brush to begin with.

*Dr. Justin D. Towner, Memphis, Tennessee*—It seems to me, the first thing we must consider is the fact that the tooth brush is not a mop; neither is it a broom. It is a series of toothpicks, and it should be used as such.

The next thing I would suggest is that when we refer to brushing the "gums and teeth," we should include the gingivae if we are going to adhere strictly to a definition of anatomical structures in our discussion—gums, gingivae and teeth.

In reference to the number of times the teeth should be brushed, I would suggest that in the morning after rising the entire mouth should be cleaned. That means the buccal membrane, the tongue, all soft tissues, as well as the hard structures, especial attention being paid to the investment tissues of the teeth. After each meal the mouth should be thoroughly cleaned and before retiring—brushing should be supplemented with the intelligent use of tape. When the tape is used intelligently, the teeth can be kept clean provided, as Dr. James has suggested, the periodontist puts the mouth in a proper condition to be cleaned by the patient.

*Dr. Arthur H. Merritt, New York City*—In a matter of such importance as this we should make haste slowly. I am perfectly confident, and I know you will agree with me, that it is impossible to devise any method that will meet with the approval of everybody, and I do not expect such a method to be adopted. There may be changes in the report, the correction of which would add to its value, and I know Dr. Hayden and the other members of the committee wish to make the report as perfect as possible. I hope that this motion will not be adopted. I would like to see the matter referred back to the council for further consideration.

Gillette Hayden, D.D.S., Chairman,
Austiin F. James, D.D.S.,
Andrew J. McDonagh, L.D.S.,
Special Committee.

